# Differential modulation of resting-state functional connectivity between amygdala and precuneus after acute physical exertion of varying intensity: indications for a role in affective regulation

**DOI:** 10.3389/fnhum.2024.1349477

**Published:** 2024-04-04

**Authors:** Marvin Lohaus, Angelika Maurer, Neeraj Upadhyay, Marcel Daamen, Luisa Bodensohn, Judith Werkhausen, Christian Manunzio, Ursula Manunzio, Alexander Radbruch, Ulrike Attenberger, Henning Boecker

**Affiliations:** ^1^Clinical Functional Imaging Group, Department of Diagnostic and Interventional Radiology, University Hospital Bonn, Bonn, Germany; ^2^Deutsche Zentrum für Neurodegenerative Erkrankungen Bonn, Bonn, Germany; ^3^Sportsmedicine, Department of Paediatric Cardiology, University Hospital Bonn, Bonn, Germany; ^4^Department of Neuroradiology, University Hospital Bonn, Bonn, Germany; ^5^Department of Diagnostic and Interventional Radiology, University Hospital Bonn, Bonn, Germany

**Keywords:** acute exercise, rs-fMRI, functional connectivity, affective regulation, amygdala, emotion, major depressive disorder, default mode network

## Abstract

**Introduction:**

Physical activity influences psychological well-being. This study aimed to determine the impact of exercise intensity on psychological well-being and alterations in emotion-related brain functional connectivity (FC).

**Methods:**

Twenty young, healthy, trained athletes performed a low- and high-intensity interval exercise (LIIE and HIIE) as well as a control condition in a within-subject crossover design. Before and after each condition, Positive And Negative Affect Scale (PANAS) was assessed as well as resting-state functional MRI (rs-fMRI). Voxel-wise FC was examined for bilateral amygdala seed region to whole-brain and emotion-related anatomical regions (e.g., insula, temporal pole, precuneus). Data analyses were performed using linear mixed-effect models with fixed factors condition and time.

**Results:**

The PANAS Positive Affect scale showed a significant increase after LIIE and HIIE and a significant reduction in Negative Affect after the control condition. In rs-fMRI, no significant condition-by-time interactions were observed between the amygdala and whole brain. Amygdala-precuneus FC analysis showed an interaction effect, suggesting reduced post-exercise anticorrelation after the control condition, but stable, or even slightly enhanced anticorrelation for the exercise conditions, especially HIIE.

**Discussion:**

In conclusion, both LIIE and HIIE had positive effects on mood and concomitant effects on amygdala-precuneus FC, particularly after HIIE. Although no significant correlations were found between amygdala-precuneus FC and PANAS, results should be discussed in the context of affective disorders in whom abnormal amygdala-precuneus FC has been observed.

## Introduction

Physical activity (PA) is known as one of the most efficient mood-regulating strategies (Thayer et al., 1994). The underlying mechanisms of affective changes triggered by PA have been investigated in animal models and a growing number of human studies (Basso and Suzuki, 2017). Importantly, from a clinical perspective, meta-analyses have confirmed that PA can significantly reduce states of anxiety and depression in patients with affective disorders (Ensari et al., 2015; Rebar et al., 2015).

Neuroanatomical structures involved in affective processing typically include the amygdala, the insula, the ventromedial prefrontal cortex (vmPFC), and the anterior cingulate cortex (Phan et al., 2002; Janak and Tye, 2015). The amygdala is a subcortical hub region of the neural circuitry critical for emotional reactivity (Gallagher and Chiba, 1996) and contemporary theories of emotion highlight its role in the evaluation and integration of different sensory inputs (Šimić et al., 2021). Concerning the processing of basic emotions, activation likelihood estimation meta-analyses of functional neuroimaging studies concluded that the amygdala reacts in particular to fearful stimuli (Vytal and Hamann, 2010), however, other studies have indicated responsiveness also to positive emotions (Costafreda et al., 2008). Clinically, functional alterations of the amygdala are assumed to play an important role in affective disorders, and there is growing interest in using magnetic resonance imaging (MRI) -derived biomarkers from both task-based and resting-state functional MRI (rs-fMRI) to track the modulatory influence of pharmacological, but also behavioral interventions in these patient groups (Malejko et al., 2017; Kotoula et al., 2023). However, current knowledge on the acute and chronic effects of exercise on the function of affect-related brain networks is still limited.

Previous imaging studies have investigated the effects of PA on amygdala resting-state functional connectivity (FC) which is estimated from the temporal covariation of brain activity fluctuations in the amygdala and other brain regions, as measured with BOLD (blood oxygenation level-dependent) rs-fMRI time series (e.g., van den Heuvel and Pol, 2010): Examining late adolescent individuals in a between-subject design, it was shown that moderate treadmill running leads to a post-acute reduction in negative affect while rs-fMRI analyses identified increased FC between the right amygdala and right orbital frontal cortex (Ge et al., 2021). Furthermore, our previous work showed in a within-subject crossover design that acute positive mood changes occurred immediately after both aerobic and anaerobic exercise, while anaerobic exercise resulted in differentially increased FC between the amygdala and insula compared to aerobic exercise (Schmitt et al., 2020). Notably, this FC change correlated with the positive mood changes found, supporting the hypothesis that increased amygdala-insula connectivity is associated with a positive change in mood in an exercise intensity-dependent manner.

Whereas these findings suggest that exercise can influence the functional coupling between the amygdala and other regions within the typical emotion-related networks, there may also be some interplay with other functional brain networks. For instance, it has been proposed that the default mode network (DMN), which includes overlapping medial frontal areas, but also posterior cingulate and precuneus, angular and mediotemporal regions (Buckner et al., 2008; Andrews-Hanna et al., 2010), is also important for constructing emotional experiences (Satpute and Lindquist, 2019). Recently, our lab has examined the longitudinal effects of aerobic exercise (three times per week over 6 months) and demonstrated increased positive FC with the left temporal pole, as well as increased anticorrelation between the amygdala and the precuneus over time in the intervention group (Maurer et al., 2022), a region that has been implicated in emotional regulation strategies via attentional deployment (Ferri et al., 2016). While this finding would support long-term adaptations of amygdala-DMN interactions, a recent task fMRI study (Kommula et al., 2023) in older adults also observed a reduced activation of the precuneus region during the visual presentation of positive emotional stimuli after 30 min of a continuous moderate-to-vigorous intensity exercise bout. Further, a study in young healthy trained men revealed reduced activity of the precuneus during the presentation of fearful faces after 30 min low-intensity continuous exercise (Schmitt et al., 2019). While not addressing amygdala connectivity directly, these studies provide indirect evidence for acute exercise-induced modulations in DMN (and specifically, precuneus function) in the emotional context.

In recent years, high-intensity interval training (HIIT) schedules have gained attention. They seem to produce similar fitness improvements and health benefits as traditional continuous training schedules, but may be especially attractive for certain audiences due to their reduced time requirements per training session (Engel et al., 2018; Martland et al., 2022). Since HIIT schedules consist of repeated, brief bursts of high-intensity performance (typically reaching 85% maximal heart rate or above) interspersed with recovery periods of low-intensity exercise or rest (Martland et al., 2022), the acute physiological responses and their subjective perception may differ from continuous training schedules. For example, a recent study (Dierkes et al., 2021) comparing the affective response for a HIIT training with a moderate-intensity and a high-intensity continuous training regimen observed similar mood decreases during both high-intensity exercise schedules (which is consistent with aversive interoceptive experiences due to exercise strain), but an “affective rebound” immediately after exercise, which was more pronounced for the HIIT condition, which suggests that the short intervals of severe load were not sufficiently long to disrupt homeostasis into the post-acute phase. In general, HIIT seems to have similar (but maybe less pronounced) post-acute effects on positive mood like continuous training schedules (Oliveira et al., 2018; Niven et al., 2021). However, the underlying brain functional alterations (e.g., of resting-state amygdala FC) may also differ from earlier neuroimaging studies using continuous moderate- or high-intensity training schedules (Schmitt et al., 2020; Ge et al., 2021).

Therefore, the present study aimed to examine the post-acute effect of interval exercise schedules on the subjective affective experience and underlying changes in the functional interaction between the amygdala (as a core region of affective processing) and other brain regions, as measured by resting-state FC. The study was conceptualized as a within-subject crossover design with a low- and high-intensity interval exercise (LIIE and HIIE) and a control condition, with pre- and post-exercise examinations of subjective affect and resting-state brain activity. This setting allowed us to investigate the effects of two exercise sessions of differing intensities and to compare these to a control condition. We hypothesized (i) a shift in the self-evaluations toward positive affect after completion of the two exercise conditions, similar to previous work (Schmitt et al., 2020). Moreover, we expected an intensity-dependent modulation of FC for the amygdala seed region: Following previous observations from continuous exercise schedules (Schmitt et al., 2020), we especially anticipated (ii) post-acute increases of amygdala-insular FC for the high-intensity condition. Based on previous evidence from a longitudinal exercise study (Maurer et al., 2022), we were also interested (iii) whether the training schedules modulate FC between the amygdala and the precuneus [see also: (Schmitt et al., 2019; Kommula et al., 2023)] and the left temporal pole, respectively. Finally, we examined whether (iv) the exercise-related changes in brain FC were associated with the subjective affect measures.

## Materials and methods

This study, referred to as BEACON (“Bicycling effects on affect and cognition in neuroscience”) was designed to investigate the acute effects of exercise bouts on brain functional networks. BEACON has a focus on affective, memory, and executive processing and aims to uncover the respective acute changes in brain networks induced by exercise sessions of varying intensity, using a within-subject crossover design with three experimental conditions: a HIIE, a LIIE, and a passive resting condition. In the current paper, we focused on exercise-induced changes of amygdala FC, as derived from resting-state fMRI examinations, and their relationship to modulations in subjective affective states, as measured via affect questionnaires, which were collected before and after each experimental condition. Other manuscripts will concentrate on other aspects of the study. Therefore, description and statistical analyses of some background data (e.g., demographical and exercise physiological parameters) are also reported in related papers (e.g., Boecker et al., 2024, preprint).

The study was approved by the local ethics committee of the University Hospital Bonn (Ethics Committee at the Medical Faculty of the Rheinische Friedrich-Wilhelms-Universität Bonn: Nr. 358/19), according to national legislation and the Declaration of Helsinki.

### Participants

Well-trained right-handed male cyclists between the ages of 20 and 35 years were recruited through local cycling and triathlon clubs, by distributing flyers at the local university clinic, and through social media. Exclusion criteria were current and/or previous severe psychiatric, neurologic, or cardiovascular diseases, and a VO_2*max*_ (maximum oxygen uptake) value lower 55 ml/min/kg. MRI-specific exclusion criteria were claustrophobia, non-removable metals/implants, tattoos exceeding a certain size, or other reasons not to undergo MRI.

### Experimental procedure

Participants were informed about the content of the study, and written informed consent was obtained after a detailed explanation of all tests, potential discomforts, risks, and procedures employed in the investigation.

Before entering the intervention sessions, participants completed a preparatory phase which included the collection of general background information, a sports medicine examination, and performance diagnostics. A sociodemographic questionnaire and vocabulary test were used to assess descriptive characteristics such as age, educational level, and estimated verbal intelligence. The International Physical Activity Questionnaire (IPAQ, Booth, 2000) was used to record the participants’ daily physical activity. Handedness was assessed using the Edinburgh-Handedness-Inventory (EHI, Oldfield, 1971). To rule out possible acute or previous depressive or other psychiatric disorders, participants answered the Mini International Neuropsychiatric Interview (Sheehan et al., 1998) and the Beck Depression Inventory (BDI, Hautzinger et al., 1994). The BDI is a 21-item questionnaire to quantify the severity of depressive symptoms. The BDI was used for sample characterization only and to exclude depressive symptoms over the course of the study. Values between 0–10 indicate ‘no depression’. Moreover, the Trait Scale of the State-Trait Anxiety Inventory (STAI, Spielberger, 1983) and a substance use questionnaire were collected.

The sports medical examination was performed on a separate day and consisted of a detailed medical history, a 12-lead resting electrocardiogram, auscultation of the heart and lungs, and transthoracic echocardiography if necessary.

For performance diagnostics, the participants’ personal bicycles were mounted on a cycling ergometer (Cyclus2, RBM Elektronik-Automation GmbH, Leipzig, Germany), analogous to the later experimental procedures. Subsequently, a step test was performed according to the protocol of the German Cycling Federation including spiroergometry. The protocol started with a 100-watt (W) load and was increased by 20W every 3 min until the subjective exhaustion of the participant. During the protocol, the participant had to maintain a cadence of at least 80 revolutions per minute. In addition to the power output, heart rate (HR), blood pressure, and respiratory gases were continuously recorded (Cortex meta-analyzer 3B, Leipzig, Germany, and Polar Electro Oy, Kempele, Finland). A 12-lead exercise electrocardiogram (Cardio 100 USB, Ergoline GmbH, Bitz, Germany) was also acquired. Lactate was collected from the participants’ earlobes at the beginning, after each step, and at the end of the procedure. Blood samples were then amperometric-enzymatically analyzed using EBIOplus (EKF Diagnostic Sales, Magdeburg, Germany). At the same time point, perception of exertion was acquired using the RPE scale (rated perceived exertion, scale 6 to 20) by Borg (1998). The performance diagnostics aimed to determine the VO_2*max*_ and the lactate thresholds D_*max*_ and ‘first rise’ of each individual. The data obtained (D_*max*_ and ‘first rise’) were then used to determine the respective individual exertion values for the subsequent experimental interventions. To take part in the interventional sessions, participants had to perform with a VO_2*max*_ of at least 55 ml/min/kg (DePauw et al., 2013). This ensured a high fitness level (Chang et al., 2013) for all subjects.

The three interventional sessions per participant were performed in randomized order on separate days and at least 7 days apart but within an 8-week period. They were identical in structure, except that the first intervention session was complemented by prior habituation in a mock scanner and additional structural MRI sequences (to exclude incipient findings suggestive of brain pathology) after completion of the experimental part.

Each intervention day began with the collection of a custom questionnaire on current sleep quality and duration, alcohol and caffeine consumption, and the BDI. Then, pre-intervention subjective ratings of mood and anxiety were collected using the Positive And Negative Affect Scale (PANAS, Krohne et al., 1996) and the STAI-state, respectively; furthermore, the MoodMeter (Kleinert, 2006; Wollseiffen et al., 2016) was acquired for assessment of the currently experienced bodily state. Further tests on executive function [Attention Network Test (Weaver et al., 2013)] and memory [Mnemonic Similarity Task (Stark et al., 2019)] were carried out as part of this study, but are not analyzed in this publication. The tests were followed by T1-weighted (T1w), fieldmap, and rs-fMRI measurements. Subsequently, participants underwent one of three interventions: (i) control condition (the participant only sat on a bicycle without any physical activity); (ii) LIIE (4 * 4 min load at 100% first rise (W) with 3 min active recovery at 90% of first rise); or (iii) HIIE (4 * 4 min load at 110% D_*max*_ (W) with 3 min active recovery at 60% of D_*max*_). The two exercise conditions consisted of a 10-min warm-up (1.5 W/kg body weight). This was followed by 4 alternating load and active recovery phases. The condition ended with a 5-min cool-down period (<1.5 W/kg body weight). HR was monitored continuously during the procedure (HR_*int*_). In addition, the participant’s blood pressure was measured before the start and after the end of the exercise intervention. In addition to a resting lactate measurement before the intervention, lactate and RPE were recorded at the end of each interval.

**FIGURE 1:**
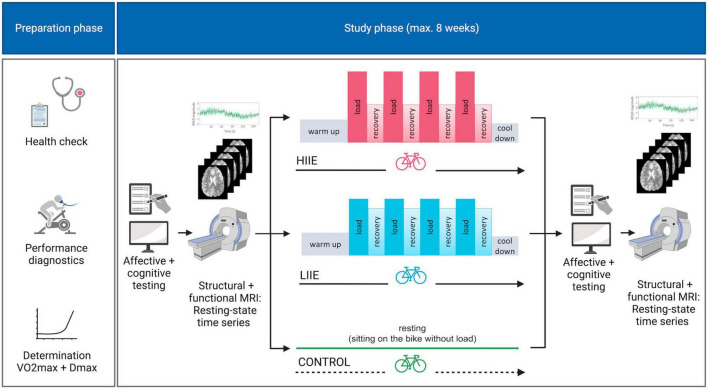
Study design. HIIE, high intensity interval exercise; LIIE, low intensity interval exercise. Created with BioRender.com.

Post-intervention data collection (mood and anxiety scores, MRI) was structured in the same way as the pre-intervention. For complete study design see Figure 1.

### Mood questionnaires

The PANAS is an instrument for assessing mood states. The questionnaire consists of a total of 20 adjectives, 10 of which can be assigned to the dimension of Positive Affect and 10 to the dimension of Negative Affect (Krohne et al., 1996), each rated on a 5-point Likert scale ranging from 1 “not at all” to 5 “very much”. This allows for a global dimensional assessment of pleasure and displeasure which are assumed to be partially separable on the brain level (Norris et al., 2010), and may therefore be influenced by acute exercise in a differential fashion.

In addition to the general assessment of Negative Affect via PANAS, we also collected specific information regarding anxiety using the STAI, which is thought to show a positive association with amygdala reactivity to aversive stimuli (Etkin et al., 2004; Chen et al., 2020). The STAI is a questionnaire for capturing the state and trait anxiety index. It consists of two scales with 20 items each. One is used to measure the intensity of current anxiety sensations (state-anxiety) and the other one to obtain anxiety as a personal trait (trait-anxiety). Items can be rated on a 4-point Likert scale ranging from 1 “not at all” to 4 “very much”. The trait-anxiety questionnaire was administered only once at the beginning of the preparation phase, the state-anxiety scale was collected before and after each intervention.

The MoodMeter is a questionnaire for the assessment of the currently experienced bodily state (Kleinert, 2006; Wollseiffen et al., 2016). It consists of three main dimensions: physical state, psychological strain, and motivation. It consists of 32 adjectives which are ranked on a scale from 0 (not at all) to 5 (completely). To account for exhaustion due to the exercise conditions and its possible influence on affective processing, the respective subscale (consisting of the items “feeble” and ‘drowsy’) from the psychological strain dimension was inserted as a nuisance variable in the statistical analyses.

### Data acquisition

All MRI scans (in total six sessions) were acquired on a 3T MRI scanner (Ingenia Elition, Philips Healthcare, Best, the Netherlands) with a 32-channel head coil at the Parent-Child Centre of the University Hospital Bonn, Germany.

Each session consisted of the acquisition of a T1w image, a fieldmap, and a rs-fMRI sequence. For rs-fMRI, the light in the examination room was switched off and participants were instructed to close their eyes, not to think of anything in particular, and to stay awake. Rs-fMRI data were acquired using a T2*-weighted gradient-echo, echo-planar imaging (EPI) sequence with 585 volumes and the following specifications: Time of repetition (TR) = 1,020 ms, echo time (TE) = 30 ms, acquired voxel size = 2.5 × 2.5 × 2.5 mm, reconstructed voxel size = 2.17 × 2.17 × 2.5 mm, field of view (FOV) = 208 × 208 × 143 mm, flip angle = 52°, SENSE: 2, MB factor: 3, EPI factor: 41, matrix: 84 × 82, slices: 51, scan order: FH (ascending), duration: 10:04 min. An index finger-mounted pulse oxymeter was used to collect cardiac data (heart rate; HR_*rest*_) during the resting state fMRI scans.

The anatomical T1w sequences were acquired with the following specifications: slice orientation: sagittal, sequence type: 3D FFE, acquisition matrix: 356 × 356, acquired voxel size: 0.7 × 0.7 × 0.7 mm, reconstructed voxel size: 0.49 × 0.49 × 0.57 mm, FOV: 250 × 250 × 190 mm, TR: 10 ms, TE: 4.7 ms, flip angle: 8°, total scan duration: 6:19 min.

Field mapping was performed with the following parameters: TR = 650 ms, TE = 7 ms, acquired voxel size: 3.75 × 3.75 × 4 mm, flip angle: 80°.

At the end of the first examination day additional T2w, 3D FLAIR, and diffusion tensor imaging sequences were acquired post experimental procedure.

#### Data preprocessing

Data quality was reviewed using MRIQC (Esteban et al., 2017), which generates a report consisting of image quality metrics and visualization of the sequences (images). The report was used to exclude data sets with MR related artifacts (e.g., signal loss, inadequate brain coverage, ring artifacts, etc.).

Preprocessing was performed using the *fMRIPrep* toolbox 20.2.6 (Esteban et al., 2018) which is based on *Nipype* 1.7.0 (Gorgolewski et al., 2011). For details of the pipeline see Supplementary Data. Further analysis of data quality was done after fmriprep by the following metrics: derivate of root mean square variance over voxels (DVARS) and framewise displacement (FD). DVARS reflects the rate of change of the BOLD signal across the whole brain at each frame of data. FD quantifies the head movement of the respective frames and was limited to a threshold below 0.20 mm. The individual subjects were excluded from the study if the respective frames FD value of > 0.20 mm was exceeded in more than 60% of the total 585 volumes after running fmriprep [limiting to at least presence of 4 min of data with low motion (FD < 0.2 mm)] (Parkes et al., 2018).

During these preprocessing steps, FD and DVARS, as well as six head motion (3 translation and 3 rotation) parameters were generated. Additionally, a set of physiological regressors was extracted using a discrete cosine filter with 128s cut-off to allow for component-based noise correction [*CompCor*, (Behzadi et al., 2007)]: temporal (tCompCor) and anatomical (aCompCor). We expanded the six head motion parameters using temporal derivatives to obtain twelve motion regressors. We opted 12 regressors for motion in order to increase the degree of freedoms after denoising because earlier comparative study reported almost similar QC-FC motion relationship after correcting for 12 or 24 motion parameters with acompcor approach (Parkes et al., 2018). Denoising of the preprocessed data was performed by regressing out the acompcor components (5 CSF and 5 WM), the 12 motion parameters as well as 8 cosine filter components for highpass filtering. For this purpose, we used the 3dTproject function from the AFNI library^1^ to perform regression of the noise, highpass filtering and smoothing (4 mm of FWHM) in one single step.

#### Amygdala seed definition and connectivity analysis

Bilateral amygdala regions of interest (ROIs) were created by using individual masks generated from the freesurfer segmentation outputs within the fmriprep pipeline. We checked visually how all FreeSurfer-based amygdala parcellations overlaid upon the respective co-registered mean EPI in each subject per exercise session, to avoid that extracted signal time courses from these seed regions contained noise from adjacent brain regions, non-brain voxels, or signal dropouts. Then, dual regression was performed to create spatiotemporal maps of covarying activity pre- and post-conditions (low, high, control).

### Statistical analysis

#### Exercise interventions

The collected data of each exercise intervention (HR_*int*_, lactate concentration, and RPE) were analyzed using Bonferroni-corrected paired t-tests. Effect sizes were reported as Cohen’s d and significance was considered at *p* < 0.05. This analysis was performed to prove that the interventions differed in their physiological load.

#### Questionnaires and physiological data

Statistical analysis of the behavioral questionnaires (PANAS, STAI state) as well as physiological data during the MRI (HR_*rest*_) was carried out with IBM SPSS Statistics (version 29.0.0.0). To test for significant changes due to the experimental factors, while also controlling for the potential variations due to subject- or session-specific background influences, a linear mixed effects (LME) model with fixed factors time (pre/post exercise) and condition (control, LIIE, HIIE) and a random intercept was performed, with age, VO_2*max*_, exhaustion, and HR_*rest*_ as continuous nuisance covariates. For further evaluation, *post hoc* Bonferroni-corrected paired t-tests were performed. Effect sizes were reported as Cohen’s d. Significance was considered at an adjusted *p* < 0.05 for the comparisons of interest (pre vs post comparisons per condition as well as three comparisons between the deltas.

#### Imaging data

The same LME model (as used for the behavioral and physiological data) was used to analyze the rs-fMRI data by implementing the 3dLMEr function^2^ in the AFNI toolbox. All 3dLME analyses were performed using a grey matter mask (derived from standard MNI152NLin2009cAsym image) to constrain the findings to grey matter regions. In addition to a whole-brain approach, further analyses were constrained to regions that were identified in earlier investigations, i.e., insula (Schmitt et al., 2020), precuneus, and left temporal pole (Maurer et al., 2022). The anatomical regions were defined by the Desikan-Killiany atlas in FreeSurfer (Desikan et al., 2006).

Following our hypotheses, we interrogated significant condition by time interactions. Multiple comparison correction was applied using the cluster-correction method (as implemented in AFNI 3DClustSim (Forman et al., 1995) including the newest approach for estimating a non-Gaussian spatial autocorrelation function (ACF) that leads to reduced false positive rates (Cox et al., 2017). Clusters were considered significant at a cluster-defining threshold of *p* < 0.005 (uncorrected) and an alpha level < 0.05.

#### Correlation analysis

For each condition, the post- minus pre-intervention changes for the PANAS scales were correlated with the respective changes in beta values within the significant clusters derived from the functional MRI analysis. Pearson correlation was used and significance was considered at a *p* < 0.05.

## Results

### Participants

A total of 29 participants were included in the study. During the course of the study, 9 participants had to be excluded: three did not reach the required VO_2*max*_ of at least 55 ml/min/kg in the performance diagnostics, four were excluded for medical reasons (induced by private activities) which occurred during the course of the study, and one was excluded due to MRI quality issues (showing more than 60% acquired volumes corrupted in three sessions out of six due to motion). For another participant usability of the resting state fMRI data was unclear, as the participant reported falling asleep during data acquisition. Thus, a total of 20 subjects were included in the final data analysis. For the IPAQ, 19 of the 20 participants reached the categorization “highly active.” Only one participant was categorized as “medium active.” The background characteristics of the subjects are summarized in Table 1.

**TABLE 1:** Characteristics of the subjects.

Variable	Mean ± standard deviation (*N* = 20)
Age (years)	27.3 ± 3.6
Height (cm)	181.6 ± 6.3
Weight (kg)	76.3 ± 6.5
BMI (kg/m^2^)	23.1 ± 1.1
VO_2max_ (ml/min/kg)	58.5 ± 3.5
Education (years)	18.9 ± 2.1
BDI	1.6 ± 1.5
STAI trait	28.7 ± 3.5
EHI	76.9 ± 22.4
WST IQ	108.1 ± 5.8

BDI, Beck Depression Inventory; BMI, Body-Mass-Index; EHI, Edinburgh-Handedness Inventory; STAI, State-Trait-Anxiety Inventory; VO_2max_, maximum oxygen uptake; WST, Wortschatztest (German vocabulary test).

### Exercise interventions

The average interval between visit 1 and visit 2 was 2.11 (± 1.49) weeks, while the average interval between visit 2 and 3 was 1.59 (± 1.01) weeks. Statistical analysis revealed no significant difference [pairwise t-test: *t*(19) = 1.14, *p* = 0.268], hence not suggesting any relevant influence of the interval between the conditions. Statistics of the variables HR_*int*_, lactate concentration, and RPE exercise parameters can be found in the Supplementary Tables 1–3. In addition, the calculated and performed loads (power output) are summarized in Supplementary Table 4 for each subject. In both, the HIIE and the LIIE intervention, individuals were successful in exercising within the required power output.

### Questionnaires

#### PANAS

For the PANAS Positive Affect scale, the LME model showed a significant main effect of time [*F*(1,94.0) = 35.9, *p* < 0.001] and condition [*F*(2,94.4) = 15.7, *p* < 0.001]. A significant time x condition interaction effect [*F*(2,94.4) = 12.6, *p* < 0.001] was also observed. The covariates age, VO_2*max*_, exhaustion, and HR_*rest*_ showed no significant effects.

**FIGURE 2:**
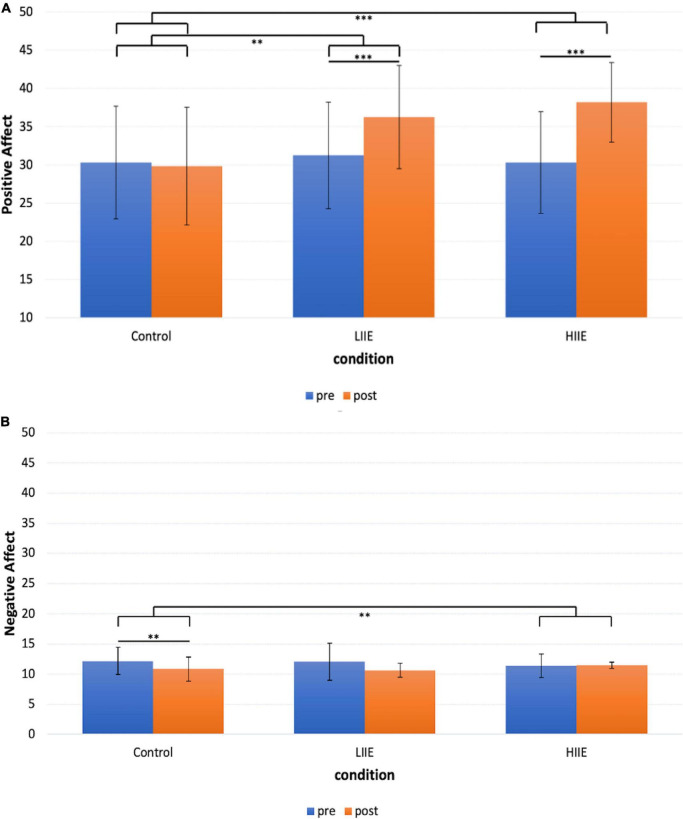
Representation of the mean ± standard deviation (SD) of the PANAS for each condition and timepoint. **(A)** PANAS Positive Affect scale; **(B)** PANAS Negative Affect scale. **p < 0.01, ***p < 0.001.

*Post hoc* pairwise comparisons within conditions (pre versus post) showed a significant increase in positive mood after the LIIE [*t*(19) = 5.7, *p* < 0.001, *d* = 1.3] and the HIIE [*t*(19) = 6.4, *p* < 0.001, *d* = 1.4]. After the control condition, no significant change occurred [*t*(19) = −0.4, *p* = 1.000, *d* = −0.1]. When comparing the changes (deltas of post minus pre) of the three conditions with each other, the LIIE [*t*(19) = 3.4, *p* = 0.003, *d* = 0.8] and the HIIE [*t*(19) = 7.1, *p* < 0.001, *d* = 1.6] showed significant increases compared to the control condition. However, there was a trend showing a stronger increase in positive affect after the HIIE compared to the LIIE condition [*t*(19) = −1.9, *p* = 0.073, *d* = −0.4]. The data are presented in Figure 2A.

For the PANAS Negative Affect scale, the LME model showed a significant main effect of time [*F*(1,95.0) = 7.8, *p* = 0.006], no significant main effect of condition [*F*(2,95.6) = 0.007, *p* = 0.9] but a significant time x condition interaction [*F*(2,95.6) = 4.5, *p* = 0.014]. The covariates age, VO_2*max*_, exhaustion, and HR_*rest*_ showed no significant effects.

*Post hoc* pairwise comparisons within conditions (pre versus post) revealed a significant decrease in negative affect after the control condition [*t*(19) = 3.9, *p* = 0.003, *d* = 0.9], and no significant changes after the LIIE [*t*(19) = 2.4, *p* = 0.075, *d* = 0.5] and the HIIE [*t*(19) = −0.4, *p* = 1.000, *d* = −0.1] condition. When comparing the changes (delta of post minus pre) of the three conditions with each other, only the HIIE condition differed significantly from the control condition [*t*(19) = −3.5, *p* = 0.008, *d* = −0.8]. No significant differences were found between the LIIE and the control condition [*t*(19) = 0.2, *p* = 1.000, *d* = 0.04], as well as between the HIIE and the LIIE condition [*t*(19) = −2.4, *p* = 0.066, *d* = −0.6]. The data are presented in Figure 2B.

#### STAI

For the STAI, the LME Model revealed no significant main effect of time [*F*(1,94.5) = 1.6, *p* = 0.208] and no significant main effect of condition [*F*(2,95.2) = 0.8, *p* = 0.437]. Further, no significant time x condition interaction could be obtained [*F*(2,95.1) = 1.0, *p* = 0.356]. The covariates age, VO_2*max*_, HR_*rest*_, and exhaustion showed no significant effects. The data are presented in the Supplementary Table 5.

### MRI physiological data (HR_*rest*_)

The results of the LME model showed a significant main effect of condition [*F*(2,95) = 10.31, *p* < 0.001], a main effect of time [*F*(1,95) = 4.30, *p* = 0.041], and a significant time × condition interaction [*F*(2,95) = 12.20, *p* < 0.001] for the variable HR_*rest*_.

Results of the *post hoc* tests are presented in Tables 2, 3.

**TABLE 2:** Statistics of paired t-tests within condition (pre versus post) for the HR_rest_.

Condition	Pre (M ± SD)	Post (M ± SD)	df	*T*-value	*p*-value	Cohens d
HIIE	52.03 ± 6.64 bpm	58.99 ± 6.95 bpm	19	−4.72	<0.001	−1.054
LIIE	52.45 ± 7.71 bpm	54.77 ± 9.49 bpm	19	−1.65	0.696	−0.368
Control	52.49 ± 8.37 bpm	48.73 ± 9.01 bpm	19	5.38	<0.001	1.202

df, number of degrees of freedom; M, mean; *p*-value, Bonferroni-corrected *p*-value; SD, standard deviation.

**TABLE 3:** Statistics of paired t-tests between conditions (deltas post minus pre) for the HR_rest_.

Delta (M ± SD)	Delta (M ± SD)	df	*T*-value	*p*-value	Cohens d
HIIE: 6.96 ± 6.60 bpm	Control: −3.75 ± 3.12 bpm	19	6.85	<0.001	1.531
LIIE: 2.32 ± 6.30 bpm	Control: −3.75 ± 3.12 bpm	19	4.50	<0.001	1.006
HIIE: 6.96 ± 6.60 bpm	LIIE: 2.32 ± 6.30 bpm	19	2.01	0.354	0.449

df, number of degrees of freedom; M, mean; *p*-value, Bonferroni-corrected *p*-value; SD, standard deviation.

### Functional MRI

The whole-brain approach revealed no significant main effects or interactions. Turning to the pre-informed analyses with constrained regions that were identified in earlier investigations, there were no significant FC changes between the amygdala and the insula nor between the amygdala and the left temporal pole.

**FIGURE 3:**
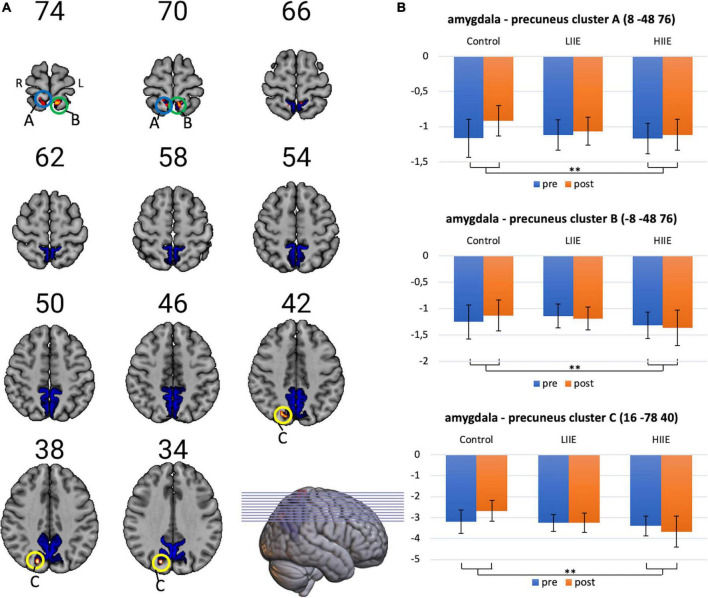
Visualization of amygdala-precuneus FC changes. **(A)** Representation of the precuneus mask, and the three clusters showing significant condition-by-time interactions (cluster-level *p* < 0.05, with voxelwise *p* < 0.005; k ≥ 20 voxels). **(B)** Corresponding mean ± standard deviation (SD) of the beta-values on the right for each timepoint and condition. Shown in radiological convention (left = right hemisphere). **p < 0.005.

However, significant condition-by-time interactions were observed when analyzing FC between the amygdala and the precuneus. *Post hoc* tests within the 3dLMEr design also showed three significant clusters [at cluster-defining threshold of *p* < 0.005 (uncorrected) and alpha-level of <0.05]. in the precuneus for the contrast between post-pre high and post-pre control. Cluster A: peak voxels: (8 −48 76), *k* = 42; Cluster B: peak voxels: (−8 −48 76), *k* = 29; Cluster C: peak voxels: (16 −78 40), *k* = 20. Findings indicate a weaker anticorrelation between the bilateral amygdala and the bilateral precuneus from pre to post in the control condition, whereas in the LIIE and HIIE conditions, no similar decrease of anticorrelation, or even stronger anticorrelation was found in the pre-to-post comparison. Additional comparisons between the conditions indicated that these interactions were driven by differential trends in the control versus HIIE condition. The significant clusters as well as the corresponding extracted beta values are shown in Figure 3.

### Correlation analyses

No significant correlations were observed between the PANAS Positive Affect and Negative Affect changes, respectively, and the changes in amygdala-precuneus FC in each cluster for each condition. The results of the correlation analyses are presented in the Supplementary Table 6.

## Discussion

This study aimed to investigate the post-acute effects of interval training schedules of different intensities on subjective emotional states and complementary modulations of amygdalar FC which may relate to these affective changes in trained athletes. The three conditions in this within-subject crossover design (control, LIIE, and HIIE) allowed intraindividual assessment of the two exercise conditions. A significant increase in positive affect ratings was found after both exercise conditions, however, not after the control condition. The increase was more pronounced after the HIIE than after the LIIE condition. A significant reduction of negative affect ratings was only observed in the control condition. There was no change in subjective state anxiety after any of the conditions. Regarding the FC analyses, the whole-brain analysis of amygdala FC changes from pre- to post-exercise revealed no significant differences between the three conditions. Region-specific follow-up analyses, informed by previous FC studies using continuous exercise schedules, indicated differences between the control and the two interval training sessions in some areas of the precuneus, which complemented earlier observations of long-term adaptations after 6-month of moderate exercise training. Given that these brain changes were not reliably associated with the reported subjective affect changes, it remains open whether they contribute to the observed exercise-related affect modulations.

Regarding the subjective affect measurements, we provide further insights into the modulation of the affective system by acute exercise bouts of differing intensities. Our work indicates that both, LIIE and HIIE bouts induce positive mood changes of similar magnitude, as measured by the PANAS Positive Affect scale. This agrees with previous studies that analyzed the acute effects of short-time exercise at low and high intensity on mood: For both intensities, a significant increase in the PANAS Positive Affect scale could be shown after the exercise compared to baseline (Bixby et al., 2001). Our research group (Schmitt et al., 2020), demonstrated that acute PA on a treadmill leads to a significant increase in the PANAS positive affect score, both, at low-intensity load (35% below lactate threshold) and high-intensity load (20% above lactate threshold). This study formed an important basis for the current investigations. We were indeed able to confirm these psychological effects in a more elaborated design which also included a resting control condition for reference, which, e.g., allowed ruling out unspecific habituation effects (Schmitt et al., 2020). Meanwhile, the present study also extended the previous work by using interval training instead of continuous training schedules, suggesting that the after-effects on positive mood were also present under these modified exercise conditions: This complements previous comparative studies which indicated that HIIT can elevate post-exercise mood, suggesting an “affective rebound” after temporary mood decline during actual exercise (Oliveira et al., 2018; Dierkes et al., 2021; Niven et al., 2021). Instead, one could have expected a carry-over of aversive bodily experiences during exercise on the post-exercise rating of negative mood states, specifically for the HIIE condition. Even though participants did not show a similar slight decline in the PANAS Negative Affect scale as seen after the control condition and (at trend level) the LIIE condition, there was no systematic increase after performing the HIIE condition. Similarly, no effects were detectable for the STAI-state scale in any of the three conditions. In general, it must be noted that the study included only healthy, trained participants without present or past affective illness, as verified by medical history, the low average performance of the STAI-trait, and repeated low BDI depression scores on the examination days, making floor effects more likely. Consistent with this observation, participants scored very low on the negative mood state questionnaires even before exercise. This would not generally preclude increases in negative mood states after high-intensity exercise: Meanwhile, the intermittent physiological load, and especially the high fitness status of the participants may have limited the impact on body homeostasis and, therefore, negative subjective experience.

Upon imaging, whole-brain voxel-wise analyses did not show systematic modulations of amygdala FC with larger brain networks, but further analyses constrained to specific brain regions that were implicated with exercise-induced changes in previous studies indicated subtle differences between the exercise conditions. While a reduced anticorrelation between the amygdala and cluster within the precuneus was observed after the control condition, notably, this effect was not observed in the exercise conditions, and, for the HIIE condition, there was even a trend for stronger anticorrelation. In general, anticorrelations of rs-fMRI fluctuations between amygdala seed regions and the posteromedial cortex areas, including the precuneus, have been observed previously (e.g., Roy et al., 2009; Cullen et al., 2014; Xiao et al., 2018). Moreover, a previous study from our group has investigated the longitudinal effects of aerobic exercise on brain plasticity and mood (Maurer et al., 2022). Young, untrained subjects were assigned to an intervention or control group. The intervention group performed fixed aerobic exercise three times per week over a period of 6 months. At intervals of 2 months, rs-fMRI was performed together with psychological tests (including PANAS). Over time, the intervention group showed a significant increase in the anti-correlation between the amygdala and precuneus (as well as increased positive FC of the amygdala with the temporal pole), which was not present in the control group (Maurer et al., 2022). Accordingly, the present results for acute exercise bouts partially resemble these previous observations (at least for the precuneus), opening the possibility that they contribute to long-term functional plasticity of connectivity patterns when performed repeatedly.

The precuneus is an important hub region of the DMN and is believed to primarily support internally oriented and self-referential thinking (Northoff et al., 2006). More recently, there was an increasing awareness that the DMN also plays an important role in constructing subjective emotional experiences (Satpute and Lindquist, 2019), and, more specifically, the precuneus has been implicated with emotional regulation strategies via attentional deployment (Ferri et al., 2016; Kommula et al., 2023). On the other hand, DMN functioning was shown to be altered in affective disorders: For example, functional MRI research suggests that major depressive disorder (MDD) in both adults and adolescents is marked by DMN hyperactivity during resting state examination (Ho et al., 2015; Kaiser et al., 2015) and that areas in the DMN are critically involved in rumination (Zhou et al., 2020). A cross-sectional rsfMRI study indicated that resting-state FC between the amygdala and the precuneus in adolescent MDD patients tended to be positive, whereas healthy controls showed the previously reported trend for anticorrelation (Cullen et al., 2014), which is also consistent with a recent meta-analysis (Tang et al., 2018) and may be related to rumination, i.e., a self-referential thought process that mainly deals with negative content (Tang et al., 2018). On the other hand, stronger DMN suppression during emotion processing appears to predict antidepressant therapeutic response in MDD: Deactivation of the precuneus and posterior cingulate cortex (PCC) during an emotion discrimination task predicted changes in Hamilton Depression Rating Scale scores after 2 weeks of treatment (Spies et al., 2017). Therefore, modulation of DMN, and also precuneus function, represents a possible target for therapeutical strategies. Considering that the HIIE condition tended to strengthen the anticorrelation between amygdala and precuneus that is observed in healthy individuals, but impaired in depressive patients, and that our previous work from a 6-month training intervention with repetitive continuous exercise bouts (Maurer et al., 2022) pointed into a similar direction, exercise may provide a low-cost, low-risk behavioral intervention strategy that deserves further research.

Meanwhile, the present results did not confirm other observations from acute exercise studies which showed increased amygdala FC with orbitofrontal regions after moderate-intensity continuous exercise bouts (Ge et al., 2021), and with insular regions after high-intensity (vs. low-intensity) continuous exercise (Schmitt et al., 2020). The limited number of relevant studies, and several methodological variations between them (between- versus within-subject designs, varying exercise intensity, fitness level, continuous vs. interval training) hinder clarification of these inconsistencies and make clear that further investigation of these context factors is needed.

Further, a correlation analysis was performed between the change scores for the PANAS scales and the beta values of the significant clusters in the amygdala-precuneus FC analysis to explore whether the observed modulations of brain function were directly related to the subjective effects of the exercise or control conditions, respectively. Following the above mentioned line of reasoning, with anticorrelation between amygdala and precuneus activity being the regular mode, one could have expected that mood improvements would go along with enhanced FC anticorrelation after treatment. Therefore, increased PANAS Positive Affect (i.e., positive change scores) should show a negative correlation (and decreased PANAS Negative Affect, i.e., negative change scores showing a positive correlation) with even more negative amygdala-precuneus FC change scores. Yet, no significant correlation could be shown. Accordingly, this line of speculation could not be verified in the current study and we have to confess that although there was a clear change toward positive mood in the exercise conditions, there was no clear evidence that this was driven by a change in the FC between amygdala and precuneus.

This study is not without limitations: First, a fairly homogeneous group of participants was included in this study, focusing on highly trained male athletes between the age of 20 and 35. This does not represent a true reflection of the general population. By selecting only young, fit and healthy athletes, the risk of cardiovascular problems during and after intense exercise should be minimized. Possibly, as noted above, the effect of exercise on positive mood is different depending on training status but this will need to be explored systematically in future studies. No female subjects were included in the study. While the literature examining menstrual cycle variations of resting-state fMRI measures provide mixed results (Hjelmervik et al., 2014; Hidalgo-Lopez et al., 2020), we aimed to minimize such nuisance factors in this pilot study. Meanwhile, future studies should explicitly explore potential sexual dimorphic effects, as they may be important moderators for beneficial exercise effects (Barha et al., 2019). In addition, the present findings, and also some earlier neuroimaging studies examining the acute effects of exercise (e.g., Weng et al., 2016) are limited by relatively limited sample sizes: A larger number of subjects (and hence, statistical power) would allow a more reliable evaluation of some observed effects that currently only appear on a trend level. In conclusion, this study was able to confirm the previous state of research that acute exercise has a positive effect on mood, as evidenced by the significant increase in the PANAS positive score after the exercise conditions. Our most interesting finding regarding the rs-fMRI analyses was the alteration of amygdalar-precuneal FC toward a stronger anticorrelation after HIIE relative to the control condition. The FC between amygdala and precuneus is important because it is not only modulated by exercise but is also pathologically altered in affective disorders like MDD. Although we did not find any significant correlations between the FC and the changes in mood, these findings lay the foundation for further clinical studies in patients with affective disorders, for whom treatment supplementation with exercise has been shown to prove beneficial and where the underlying network modulations, in particular the effects of acute exercise on amygdala-precuneus FC, will need to be further disentangled.

## Data availability statement

The original contributions presented in this study are included in the article/Supplementary material, further inquiries can be directed to the corresponding author.

## Ethics statement

The studies involving humans were approved by Ethics Committee at the Medical Faculty of the Rheinische Friedrich-Wilhelms-Universität Bonn. The studies were conducted in accordance with the local legislation and institutional requirements. The participants provided their written informed consent to participate in this study.

## Author contributions

ML: Data curation, Formal analysis, Investigation, Methodology, Visualization, Writing—original draft. AM: Conceptualization, Data curation, Formal analysis, Investigation, Methodology, Visualization, Writing—original draft. NU: Conceptualization, Data curation, Investigation, Methodology, Visualization, Writing—review and editing. MD: Conceptualization, Data curation, Methodology, Visualization, Writing—original draft. LB: Data curation, Formal analysis, Investigation, Writing—review and editing. JW: Data curation, Formal analysis, Investigation, Writing—review and editing. CM: Data curation, Formal analysis, Investigation, Resources, Supervision, Writing—review and editing. UM: Data curation, Investigation, Resources, Writing—review and editing. AR: Funding acquisition, Writing—review and editing. UA: Funding acquisition, Resources, Writing—review and editing. HB: Conceptualization, Investigation, Resources, Supervision, Validation, Writing—original draft.

## Footnotes

1
https://afni.nimh.nih.gov/pub/dist/doc/program_help/3dTproject.html

2
https://afni.nimh.nih.gov/pub/dist/doc/program_help/3dLMEr.html
