# Supplementary material for: Differential modulation of resting-state functional connectivity between amygdala and precuneus after acute physical exertion of varying intensity: indications for a role in affective regulation
Source: Front Hum Neurosci. 2024 Apr 4;18:1349477. doi: 10.3389/fnhum.2024.1349477 (PMC11027744; doi:10.3389/fnhum.2024.1349477)
Supplement: Supplementary file 1 [file Data_Sheet_1.PDF]

Differential Modulation of Resting-state Functional Connectivity Between Amygdala and Precuneus after Acute Physical Exertion of Varying Intensity: Indications for a Role in Affective Regulation.

Marvin Lohaus[1†], Angelika Maurer[1†], Neeraj Upadhyay[1], Marcel Daamen[2], Luisa Bodensohn[1], Judith Werkhausen[1], Christian Manunzio[3], Ursula Manunzio[3], Alexander Radbruch[4], Ulrike Attenberger[5], Henning Boecker[1*]

1 Clinical Functional Imaging Group, Department of Diagnostic and Interventional Radiology, University Hospital Bonn, Bonn, Germany

2 Deutsche Zentrum für Neurodegenerative Erkrankungen Bonn, Bonn, Germany

3 Sportsmedicine, Department of Paediatric Cardiology, University Hospital Bonn, Bonn, Germany

4 Department of Neuroradiology, University Hospital Bonn, Bonn, Germany

5 Department of Diagnostic and Interventional Radiology, University Hospital Bonn, Bonn, Germany

[†] both authors contributed equally

[*] Correspondence to:

Univ.-Prof. Dr. med. Henning Boecker (https://orcid.org/0000-0003-2346-0598)
Head of Clinical Functional Imaging Lab
Department of Diagnostic and Interventional Radiology
(Director: Prof. Ulrike Attenberger)
University Hospital Bonn
Venusberg Campus 1
53127 Bonn
Germany
Phone.: +49 (0)228 287 - 15980
Fax: +49 (0)228 287 - 14457
Email: Henning.Boecker@ukbonn.de

Detailed Description of the fMRIPrep Pipeline (Boilerplate)

The following boilerplate description was automatically generated by fmriprep with the expressed intention that users should copy and paste this text into their manuscripts without changes. It is released under CC0 license.

Preprocessing of structural and functional MRI data was performed using fMRIPrep 20.2.6 (Esteban et al., 2017, 2018; RRID:SCR_016216), which is based on Nipype 1.7.0 (Gorgolewski et al., 2011, 2018; RRID:SCR_002502).

Anatomical data preprocessing

A total of 6 T1-weighted (T1w) images were found within the input BIDS dataset. All of them were corrected for intensity non-uniformity (INU) with N4BiasFieldCorrection (Tustison et al. 2010), distributed with ANTs 2.3.3 (Avants et al., 2008; RRID:SCR_004757). The T1w-reference was then skull-stripped with a Nipype implementation of the antsBrainExtraction.sh workflow (from ANTs), using OASIS30ANTs as target template. Brain tissue segmentation of cerebrospinal fluid (CSF), white-matter (WM) and gray-matter (GM) was performed on the brain-extracted T1w using fast (FSL 5.0.9; Zhang et al., 2001; RRID:SCR_002823). A T1w-reference map was computed after registration of 6 T1w images (after INU-correction) using mri_robust_template (FreeSurfer 6.0.1; Reuter et al., 2010). Brain surfaces were reconstructed using recon-all (FreeSurfer 6.0.1; Dale et al., 1999; RRID:SCR_001847)), and the brain mask estimated previously was refined with a custom variation of the method to reconcile ANTs-derived and FreeSurfer-derived segmentations of the cortical gray-matter of Mindboggle (Klein et al., 2017; RRID:SCR_002438). Volume-based spatial normalization to two standard spaces (MNI152NLin2009cAsym, MNI152NLin6Asym) was performed through nonlinear registration with antsRegistration (ANTs 2.3.3), using brain-extracted versions of both T1w reference and the T1w template. The following templates were selected for spatial normalization: ICBM 152 Nonlinear Asymmetrical template version 2009c (Fonov et al., 2009; RRID:SCR_008796; TemplateFlow ID: MNI152NLin2009cAsym), FSL's MNI ICBM 152 non-linear 6th Generation Asymmetric Average Brain Stereotaxic Registration Model (Evans et al., 2012; RRID:SCR_002823; TemplateFlow ID: MNI152NLin6Asym).

Functional data preprocessing

For each of the 6 BOLD runs found per subject (across all tasks and sessions), the following preprocessing was performed. First, a reference volume and its skull-stripped version were generated using a custom methodology of fMRIPrep. A B0-nonuniformity map (or fieldmap) was directly measured with an MRI scheme designed with that purpose (typically, a spiral pulse sequence). The fieldmap was then co-registered to the target EPI (echo-planar imaging) reference run and converted to a displacements field map (amenable to registration tools such as ANTs) with FSL's fugue and other SDCflows tools. Based on the estimated susceptibility distortion, a corrected EPI (echo-planar imaging) reference was calculated for a more accurate co-registration with the anatomical reference. The BOLD reference was then co-registered to the T1w reference using bbregister (FreeSurfer) which implements boundary-based registration (Greve & Fischl, 2009). Co-registration was configured with six degrees of freedom. Head-motion parameters with respect to the BOLD reference (transformation matrices, and six corresponding rotation and translation parameters) are estimated before any spatiotemporal filtering using mcflirt (FSL 5.0.9; Jenkinson et al., 2002)). The BOLD time-series (including slice-timing correction when applied) were resampled onto their original, native space by applying a single, composite transform to correct for head-motion and

susceptibility distortions. These resampled BOLD time-series will be referred to as preprocessed BOLD in original space, or just preprocessed BOLD. The BOLD time-series were resampled into standard space, generating a preprocessed BOLD run in MNI152NLin2009cAsym space. First, a reference volume and its skull-stripped version were generated using a custom methodology of fMRIPrep. Automatic removal of motion artifacts using independent component analysis (ICA-AROMA; Pruim et al., 2015) was performed on the preprocessed BOLD on MNI space time-series after removal of non-steady state volumes and spatial smoothing with an isotropic, Gaussian kernel of 6mm FWHM (full-width half-maximum). Corresponding "non-aggresively" denoised runs were produced after such smoothing. Additionally, the "aggressive" noise-regressors were collected and placed in the corresponding confounds file. Several confounding time-series were calculated based on the preprocessed BOLD: framewise displacement (FD), DVARS and three region-wise global signals. FD was computed using two formulations following Power (absolute sum of relative motions; Power et al., 2014) and Jenkinson (relative root mean square displacement between affines; Jenkinson et al., 2002). FD and DVARS are calculated for each functional run, both using their implementations in Nipype (following the definitions by Power et al., 2014). The three global signals are extracted within the CSF, the WM, and the whole-brain masks. Additionally, a set of physiological regressors were extracted to allow for component-based noise correction, ((CompCor; Behzadi et al., 2007). Principal components are estimated after high-pass filtering the preprocessed BOLD time-series (using a discrete cosine filter with 128s cut-off) for the two CompCor variants: temporal (tCompCor) and anatomical (aCompCor). tCompCor components are then calculated from the top 2% variable voxels within the brain mask. For aCompCor, three probabilistic masks (CSF, WM and combined CSF+WM) are generated in anatomical space. The implementation differs from that of Behzadi et al. in that instead of eroding the masks by 2 pixels on BOLD space, the aCompCor masks are subtracted a mask of pixels that likely contain a volume fraction of GM. This mask is obtained by dilating a GM mask extracted from the FreeSurfer's aseg segmentation, and it ensures components are not extracted from voxels containing a minimal fraction of GM. Finally, these masks are resampled into BOLD space and binarized by thresholding at 0.99 (as in the original implementation). Components are also calculated separately within the WM and CSF masks. For each CompCor decomposition, the k components with the largest singular values are retained, such that the retained components' time series are sufficient to explain 50 percent of variance across the nuisance mask (CSF, WM, combined, or temporal). The remaining components are dropped from consideration. The head-motion estimates calculated in the correction step were also placed within the corresponding confounds file. The confound time series derived from head motion estimates and global signals were expanded with the inclusion of temporal derivatives and quadratic terms for each (Satterthwaite et al., 2013). Frames that exceeded a threshold of 0.5 mm FD or 1.5 standardised DVARS were annotated as motion outliers. All resamplings can be performed with a single interpolation step by composing all the pertinent transformations (i.e. head-motion transform matrices, susceptibility distortion correction when available, and co-registrations to anatomical and output spaces). Gridded (volumetric) resamplings were performed using antsApplyTransforms (ANTs), configured with Lanczos interpolation to minimize the smoothing effects of other kernels (Lanczos, 1964). Non-gridded (surface) resamplings were performed using mri_vol2surf (FreeSurfer).

Many internal operations of fMRIPrep use Nilearn 0.6.2 (Abraham et al., 2014; RRID:SCR_001362), mostly within the functional processing workflow. For more details of the pipeline, see the section corresponding to workflows in fMRIPrep's documentation.

Copyright Waiver

The above boilerplate text was automatically generated by fMRIPrep with the express intention that users should copy and paste this text into their manuscripts unchanged. It is released under the CC0 license

Table S1: Results of statistical analysis (paired t-test) of HR_{int}.

HR_{int} [bpm]		HIIE (M ± SD)	LIIE (M ± SD)	p-value	T-value	df	Cohens d
Interval 1	load	167 ± 9	138 ± 10	<0.001	13.261	19	2.965
	recovery	139 ± 10	136 ± 9	0.669	1.436	19	0.321
Interval 2	load	176 ± 7	144 ± 11	<0.001	11.817	19	2.642
	recovery	145 ± 9	140 ± 11	0.641	1.461	19	0.327
Interval 3	load	180 ± 7	146 ± 10	<0.001	13.693	19	3.062
	recovery	151 ± 11	142 ± 10	0.028	3.029	19	0.677
Interval 4	load	184 ± 6	148 ± 11	<0.001	13.917	19	3.112
	recovery	151 ± 11	143 ± 11	0.088	2.493	19	0.557

df: number of degrees of freedom, HIIE: high intensity interval exercise, La: lactate, LIIE: low intensity interval exercise, M: mean, p-value: Bonferroni-corrected p-value, SD: standard deviation.

Table S2: Results of statistical analysis (paired t-test) of lactate data.

La [mmol·L]		HIIE (M ± SD)	LIIE (M ± SD)	p-value	T-value	df	Cohens d
Interval 1	load	5.7 ± 1.2	1.5 ± 0.6	<0.001	20.457	19	4.574
	recovery	5.4 ± 1.6	1.4 ± 0.7	<0.001	13.754	18	3.155
Interval 2	load	7.2 ± 1.9	1.5 ± 0.7	<0.001	16.816	19	3.760
	recovery	6.5 ± 2.4	1.4 ± 0.6	<0.001	10.774	19	2.409
Interval 3	load	8.0 ± 2.4	1.4 ± 0.6	<0.001	14.126	19	3.159
	recovery	7.3 ± 2.7	1.4 ± 0.6	<0.001	10.189	18	2.338
Interval 4	load	8.6 ± 2.8	1.5 ± 0.5	<0.001	11.963	18	2.745
	recovery	8.1 ± 3.2	1.4 ± 0.6	<0.001	10.363	19	2.317

df: number of degrees of freedom, HIIE: high intensity interval exercise, La: lactate, LIIE: low intensity interval exercise, M: mean, p-value: Bonferroni-corrected p-value, SD: standard deviation.

Table S3: Results of statistical analysis (paired t-test) of RPE data.

RPE		HIIE (M ± SD)	LIIE (M ± SD)	p-value	T-value	df	Cohens d
Interval 1	load	15 ± 1	11 ± 2	<0.001	7.535	19	1.685
	recovery	10 ± 1	11 ± 2	1.000	-0.396	19	-0.089
Interval 2	load	16 ± 1	12 ± 1	<0.001	9.469	19	2.117
	recovery	11 ± 2	11 ± 2	1.000	0.954	19	0.213
Interval 3	load	16 ± 1	12 ± 1	<0.001	8.976	19	2.007
	recovery	12 ± 2	11 ± 2	0.057	2.698	19	0.603
Interval 4	load	17 ± 1	12 ± 2	<0.001	11.786	19	2.636
	recovery	12 ± 2	11 ± 2	1.000	0.879	19	0.197

df: number of degrees of freedom, HIIE: high intensity interval exercise, LIIE: low intensity interval exercise, M: mean, p-value: Bonferroni-corrected p-value, SD: standard deviation.

Table S4: Power Output of each subject in both exercise interventions.

			HIIE				LIIE			
**Subject**	**First Rise [W]**	**D_{max} [W]**	**Target_Load 110% D_{max} [W] 4*4 min**	**Load**	**Target_Recovery 60% D_{max} [W] 4*3 min**	**Recovery**	**Target_Load 100% First Rise [W] 4*4 min**	**Load**	**Target_Recovery 90% First Rise [W] 4*3 min**	**Recovery**
01	160	231	254	254 ± 5	139	139 ± 5	160	160 ± 0	144	144 ± 0
02	180	237	261	261 ± 3	142	142 ± 0	180	180 ± 0	162	162 ± 0
03	220	299	329	329 ± 5	179	179 ± 7	220	220 ± 0	198	198 ± 0
04	180	257	283	283 ± 0	154	154 ± 0	180	180 ± 1	162	162 ± 0
05	200	270	298	298 ± 3	162	162 ± 4	200	200 ± 2	180	180 ± 1
06	220	284	313	313 ± 0	170	170 ± 0	220	220 ± 1	198	198 ± 1
07*	280	340	374	-	-	-	280	280 ± 1	252	252 ± 1
08	180	246	270	270 ± 4	147	147 ± 3	180	180 ± 2	162	162 ± 0
09	240	288	317	317 ± 3	173	173 ± 4	240	240 ± 3	216	216 ± 1
10	220	280	307	307 ± 3	168	168 ± 5	220	220 ± 1	198	198 ± 1
11	200	285	313	313 ± 4	171	171 ± 4	200	200 ± 0	180	180 ± 1
12	300	345	379	379 ± 0	207	207 ± 5	300	300 ± 1	270	270 ± 1
13	220	280	308	308 ± 0	168	168 ± 4	220	220 ± 1	198	198 ± 1
14	140	222	244	244 ± 0	133	133 ± 3	140	140 ± 0	126	126 ± 0
15	180	233	256	256 ± 4	140	140 ± 3	180	180 ± 0	162	162 ± 0
16	240	297	327	327 ± 0	178	178 ± 0	240	240 ± 3	216	216 ± 1
17	200	337	371	371 ± 4	202	201 ± 12	200	200 ± 2	180	180 ± 1
18	260	330	363	363 ± 8	198	198 ± 6	260	260 ± 1	234	234 ± 1
19	180	232	255	255 ± 4	139	139 ± 5	180	180 ± 0	162	162 ± 0
20	180	240	264	264 ± 3	144	144 ± 3	180	180 ± 1	162	162 ± 0

Individual power value in W for First Rise and D_{max}, determined in performance diagnostics. Target_Load and Target_Recovery values are showing the calculated target values for the corresponding intervals during the high-intensity and low-intensity conditions. The values in the respective following column represent the

mean (M) ± standard deviation (SD) of the actual power values during the condition. * Missing values due to technical problems exporting the performance data after the condition. HIIE: high intensity interval exercise, LIIE: low intensity interval exercise.

Table S5: STAI state scores presented as mean ± standard deviation.

Condition	pre	Post
**control**	30.5 ± 6.1	29.6 ± 5.9
**LIIE**	31.0 ± 7.0	28.5 ± 4.3
**HIIE**	30.0 ± 5.9	29.6 ± 5.3

HIIE: high intensity interval exercise, LIIE: low intensity interval exercise.

Table S6: Correlation analyses between PANAS and beta-values of significant precuneus clusters.

		Positive Scale		Negative Scale	
	**condition**	**Pearson correlation coefficient r**	**p-value**	**Pearson correlation coefficient r**	**p-value**
**Cluster A**	control	0.213	0.366	-0.095	0.691
	LIIE	-0.349	0.131	0.052	0.828
	HIIE	-0.323	0.165	-0.266	0.256
**Cluster B**	control	0.262	0.264	0.161	0.497
	LIIE	-0.377	0.102	0.092	0.701
	HIIE	-0.306	0.189	-0.291	0.214
**Cluster C**	control	0.285	0.223	0.085	0.720
	LIIE	-0.243	0.302	-0.296	0.204
	HIIE	-0.358	0.122	-0.132	0.578

HIIE: high intensity interval exercise, LIIE: low intensity interval exercise.

References

Abraham, A., Pedregosa, F., Eickenberg, M., Gervais, P., Mueller, A., Kossaifi, J., Gramfort, A., Thirion, B., & Varoquaux, G. (2014). Machine learning for neuroimaging with scikit-learn. *Frontiers in Neuroinformatics*, *8*, 14. https://doi.org/10.3389/fninf.2014.00014

Avants, B. B., Epstein, C. L., Grossman, M., & Gee, J. C. (2008). Symmetric diffeomorphic image registration with cross-correlation: Evaluating automated labeling of elderly and neurodegenerative brain. *Medical Image Analysis*, *12*(1), 26–41. https://doi.org/10.1016/j.media.2007.06.004

Behzadi, Y., Restom, K., Liau, J., & Liu, T. T. (2007). A component based noise correction method (CompCor) for BOLD and perfusion based fMRI. *NeuroImage*, *37*(1), 90–101. https://doi.org/10.1016/j.neuroimage.2007.04.042

Dale, A. M., Fischl, B., & Sereno, M. I. (1999). Cortical Surface-Based Analysis I. Segmentation and Surface Reconstruction. *NeuroImage*, *9*(2), 179–194. https://doi.org/10.1006/nimg.1998.0395

Esteban, O., Birman, D., Schaer, M., Koyejo, O. O., Poldrack, R. A., & Gorgolewski, K. J. (2017). MRIQC: Advancing the automatic prediction of image quality in MRI from unseen sites. *PLoS ONE*, *12*(9), e0184661. https://doi.org/10.1371/journal.pone.0184661

Esteban, O., Markiewicz, C. J., Blair, R. W., Moodie, C. A., Isik, A. I., Erramuzpe, A., Kent, J. D., Goncalves, M., DuPre, E., Snyder, M., Oya, H., Ghosh, S. S., Wright, J., Durnez, J., Poldrack, R. A., & Gorgolewski, K. J. (2018). fMRIPrep: a robust preprocessing pipeline for functional MRI. *Nature Methods*, *16*(1), 111–116. https://doi.org/10.1038/s41592-018-0235-4

Evans, A. C., Janke, A. L., Collins, D. L., & Baillet, S. (2012). Brain templates and atlases. *NeuroImage*, *62*(2), 911–922. https://doi.org/10.1016/j.neuroimage.2012.01.024

Fonov, V., Evans, A., McKinstry, R., Almli, C., & Collins, D. (2009). Unbiased nonlinear average age-appropriate brain templates from birth to adulthood. *NeuroImage*, *47*, S102. https://doi.org/10.1016/s1053-8119(09)70884-5

Gorgolewski, K., Burns, C. D., Madison, C., Clark, D., Halchenko, Y. O., Waskom, M. L., & Ghosh, S. S. (2011). Nipype: A Flexible, Lightweight and Extensible Neuroimaging Data Processing Framework in Python. *Frontiers in Neuroinformatics*, *5*, 13. https://doi.org/10.3389/fninf.2011.00013

Gorgolewski, K., Esteban, O., Markiewicz, C., Ziegler, E., Ellis, D. G., Notter, M. P., & Jarecka, D. (2018). *Nipype*. https://zenodo.org/records/6834519

Greve, D. N., & Fischl, B. (2009). Accurate and robust brain image alignment using boundary-based registration. *NeuroImage*, *48*(1), 63–72. https://doi.org/10.1016/j.neuroimage.2009.06.060

Jenkinson, M., Bannister, P., Brady, M., & Smith, S. (2002). Improved Optimization for the Robust and Accurate Linear Registration and Motion Correction of Brain Images. *NeuroImage*, *17*(2), 825–841. https://doi.org/10.1006/nimg.2002.1132

Klein, A., Ghosh, S. S., Bao, F. S., Giard, J., Häme, Y., Stavsky, E., Lee, N., Rossa, B., Reuter, M., Neto, E. C., & Keshavan, A. (2017). Mindboggling morphometry of human brains. *PLoS Computational Biology*, *13*(2), e1005350. https://doi.org/10.1371/journal.pcbi.1005350

Lanczos, C. (1964). Evaluation of Noisy Data. *Journal of the Society for Industrial and Applied Mathematics Series B Numerical Analysis*, *1*(1), 76–85. https://doi.org/10.1137/0701007

Power, J. D., Mitra, A., Laumann, T. O., Snyder, A. Z., Schlaggar, B. L., & Petersen, S. E. (2014). Methods to detect, characterize, and remove motion artifact in resting state fMRI. *NeuroImage*, *84*, 320–341. https://doi.org/10.1016/j.neuroimage.2013.08.048

Pruim, R. H. R., Mennes, M., Rooij, D. van, Llera, A., Buitelaar, J. K., & Beckmann, C. F. (2015). ICA-AROMA: A robust ICA-based strategy for removing motion artifacts from fMRI data. *NeuroImage*, *112*, 267–277. https://doi.org/10.1016/j.neuroimage.2015.02.064

Reuter, M., Rosas, H. D., & Fischl, B. (2010). Highly accurate inverse consistent registration: A robust approach. *NeuroImage*, *53*(4), 1181–1196. https://doi.org/10.1016/j.neuroimage.2010.07.020

Satterthwaite, T. D., Elliott, M. A., Gerraty, R. T., Ruparel, K., Loughead, J., Calkins, M. E., Eickhoff, S. B., Hakonarson, H., Gur, R. C., Gur, R. E., & Wolf, D. H. (2013). An improved framework for confound regression and filtering for control of motion artifact in the preprocessing of resting-state functional connectivity data. *NeuroImage*, *64*, 240–256. https://doi.org/10.1016/j.neuroimage.2012.08.052

Zhang, Y., Brady, M., & Smith, S. (2001). Segmentation of Brain MR Images Through a Hidden Markov Random Field Model and the Expectation-Maximization Algorithm. *IEEE Transactions on Medical Imaging*, *20*(1), 45. https://doi.org/10.1109/42.906424
